# Detecting Non-Brownian Trait Evolution in Adaptive Radiations

**DOI:** 10.1371/journal.pbio.0040373

**Published:** 2006-11-07

**Authors:** Robert P Freckleton, Paul H Harvey

**Affiliations:** 1 Department of Animal and Plant Sciences, University of Sheffield, Sheffield, United Kingdom; 2 Department of Zoology, University of Oxford, Oxford, United Kingdom; University of California Berkeley, United States of America

## Abstract

Many phylogenetic comparative methods that are currently widely used in the scientific literature assume a Brownian motion model for trait evolution, but the suitability of that model is rarely tested, and a number of important factors might affect whether this model is appropriate or not. For instance, we might expect evolutionary change in adaptive radiations to be driven by the availability of ecological niches. Such evolution has been shown to produce patterns of change that are different from those modelled by the Brownian process. We applied two tests for the assumption of Brownian motion that generally have high power to reject data generated under non-Brownian niche-filling models for the evolution of traits in adaptive radiations. As a case study, we used these tests to explore the evolution of feeding adaptations in two radiations of warblers. In one case, the patterns revealed do not accord with Brownian motion but show characteristics expected under certain niche-filling models.

## Abstract 2

Two diagnostic tests reveal when traditional phylogenetic comparative methods relying on a Brownian motion model of trait evolution are not appropriate, and these can help identify underlying ecological mechanisms of adaptation.

## Introduction

Phylogenetic comparative methods are used extensively to explore patterns of correlated evolution of traits or to correct for statistical nonindependence amongst species that arises as a consequence of common ancestry [1,2]. Underlying most modern comparative methods is a model of trait evolution that specifies the expected distribution of trait values, thereby allowing robust statistical tests for the correlated evolution of traits. However if the model of trait evolution assumed by a phylogenetic comparative method is incorrect, then subsequent comparative analyses may be invalid [3].

Many comparative methods rely on the Brownian motion model of trait evolution [4], which correctly predicts that more closely related species should be more similar to each other [2,5]. Several models allied to the simple Brownian motion model have been developed and applied to comparative data that do not explicitly account for ecological processes occurring during evolution [6–8]. More recent theoretical studies have explored the performance of current comparative methods when analysing ecological niche-filling models that determine the rate and amount of trait evolution [9–11]. Both niche-filling and Brownian motion models can predict that closely related species should be more similar to each other, but may yield phylogenetically correlated patterns of trait distributions across species that are very different from each other.

Niche-filling models are likely to be of particular significance in the evolution of adaptive radiations. In adaptive radiations, speciation and trait evolution may be closely linked if ecological specialisation drives or promotes genetic divergence between populations [12]. Such mechanisms can have important consequences for trait evolution. For instance during the evolution of an adaptive radiation according to a niche-filling model, if newly occupied niches are speciated by the closest species in niche space, the difference between parent and offspring species may be expected to get smaller as more of niche space becomes occupied. Irrespective of the rate at which new species arise, the amount of trait change per speciation event would be expected to decrease as the number of species added increases. In fact, trait evolution during adaptive radiation has been shown to exhibit frequent ecological differentiation that occurs with (or shortly after) speciation [12,13]. Similarly, it has been argued that niche separation along a small number of fundamental environmental axes may be a key component of cross-species patterns of diversification in plant communities [14,15]. These are examples of patterns of trait evolution that are distinctly non-Brownian.

Because of such ecologically constrained trait evolution, statistical comparative methods that assume constant rates of evolution (trait change) through time (e.g., as in Brownian motion) may be severely compromised [10]. More importantly, reliance on a Brownian model may result in the underlying nature of trait evolution not being recognised and characterised.

The Brownian model makes a number of simple assumptions [16]. The most commonly tested assumption is that trait variance accrues as a linear function of time, which can be addressed using a simple diagnostic. Further to this, a number of previous comparative methods work by transforming data or trees onto a scale that yields the best fit to a Brownian model [4–6, 17]. Some models, such as niche-filling models, will not transform to Brownian motion, and hence this approach will fail unless it is possible to diagnose non-Brownian trait evolution.

We tested for patterns of evolution that are inconsistent with Brownian motion. Here we present two simple tests that are generally effective in detecting non-Brownian trait evolution that is consistent with certain niche-filling modes of evolution. We applied these tests to simulated and real data, highlighting that deviations from Brownian models may be indicative of underlying ecological mechanisms.

## Methods

### Niche-Filling Model

The rationale of niche-filling models is that species occupy discrete niches, which determine the values of traits required to exploit them. In the models considered to date, niches remain constant through time. For instance, the morphology of beaks of different species within a group of birds may be selected to deal with just one of an array of seed types. If a given species of bird has evolved to feed on a particular seed that does not change in size or shape, then beak morphology remains constant through evolutionary time. Evolution only proceeds when a new niche becomes available. Thus, if a new form of seed appears that is not being exploited, and if there is selection for one of the bird species to feed on this new seed, then there may follow a short period of morphological evolution and perhaps eventual speciation in order to exploit the new resource.

In the simulations described below, niches are characterised by a unique optimum of either one or two characters, modelled as a normal distribution in the case of one character, or a bivariate-normal distribution with correlation coefficient *r* in the case of two characters. We restrict ourselves to a maximum of two niche axes, although the models may be extended to consider additional axes [10]. It is envisaged that the correlation *r* will generally be strong, reflecting functional associations between traits (e.g., close relationships between bill dimensions and food size in the example given in the paragraph above), or possibly trade-offs between them.

The model we analyse was originally suggested by Price [9] and subsequently refined [10,11]. The niche space is initially empty. New niches arise sequentially and are immediately invaded. The first niche and species occupying it are assigned a position within the niche space at random according the assumed distribution of niches. Subsequent niches arise at a given rate (see below) and are also positioned at random (according to the same distribution and correlation) within niche space. A new niche that arises is invaded by that extant species whose phenotype is closest to the optimum for the vacant niche, with the following consequences: (i) closely related species occupy similar niches and have similar phenotypes and (ii) as niche space fills, new niches tend to be invaded by species ever closer to them in niche space. Variants on this basic model, e.g., with all niches being available and vacant from the start, are discussed elsewhere [11].

This model has a number of characteristics. First, the correlation between traits is different from the correlation between trait changes, because niches arise within the niche space independently of the location of other occupied niches; different models vary the rules by which species invade the niches [11]. Second, in general, the amount of evolution (i.e., the difference) between parent and daughter species is determined by the distance between new and existing niches, rather than by the gradual accumulation of variance through evolutionary time. At any point in time, the amount of evolution therefore depends on the number and distribution of extant niches. Third, the distribution of trait values is constrained by the bivariate correlation, as opposed to unconstrained models such as the Brownian model.

### Brownian Model

The conventional model of trait evolution is the Brownian (motion) model, the derivation of which is outlined in some detail elsewhere [16]. We consider the evolution of a pair of traits, represented by a vector **x**. Under the Brownian model, if *t* is the time over which the trait is evolving, then Δ**x**, the change in **x**, is a bivariate normal (BVN) random deviate:

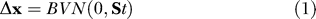

**S** is a (2 × 2) matrix containing the variances and covariances for trait changes in the case of two traits, or is a scalar in the case of one trait. The diagonals of this matrix contain the variances for the changes in each trait individually, whilst the off-diagonal elements are the covariances in trait change. After *T* units of time, **x**(*T*) is thus a bivariate normally-distributed random variate with mean **x**(0) and variance-covariance matrix **S** *T*.

From the point of view of modelling trait evolution, the key features of the Brownian model are as follows: (i) traits evolve constantly, i.e., they do not become fixed at an optimum value for each species; (ii) the evolution of all species is independent, such that potentially several species may adopt similar trait values; (iii) the values of traits are effectively unbounded (i.e., the variance in trait values grows linearly with time of evolution).

### Model of Speciation

We assume three contrasting modes of speciation for both niche-filling and Brownian models. In the niche-filling simulations, the rate of speciation is the rate at which new niches appear and are invaded, whilst in the Brownian model, the rate of speciation is the rate at which lineages split. The first model of speciation assumes that the instantaneous probability of a speciation event occurring is proportional to the number of species present. This corresponds to the familiar Yule process and generates an increasing net rate of speciation, with the intervals between the origin of new species declining through time. The second model assumes that the instantaneous probability of a speciation event is constant irrespective of the number of species present. In the third model, we assume that the instantaneous probability of a speciation event declines with the number of species present.

These three models correspond to different underlying processes that may accompany the invasion of an empty niche space. The Yule model assumes that all species are equally likely to speciate at any given time. On the other hand, a constant rate of speciation would occur when niches become available at a constant rate through time and are invaded by new species as soon as they appear. The declining rate of speciation may be thought of as modelling the initially rapid speciation that accompanies ecological diversification in the early stages of an adaptive radiation, which subsequently slows down as niches are filled and new niches become rarer.

In a true adaptive radiation not all of these scenarios are equally as likely [12]. Most likely, the rate of speciation would be expected to be initially high and slow down as the niche-space becomes filled [12]. However, here we explore all three in order to examine the sensitivity of the results obtained to tree topology.

### Phylogenetic Methodology

#### Contrasts analysis.

The starting point is the method of contrasts [1,16,17], which is a computationally simple method for fitting a Brownian motion model. We calculated phylogenetic contrasts in the following way [1] (we describe the method, which is the standard way of calculating contrasts, in detail here, because this method forms the basis for subsequent analyses):

First, beginning with a pair of adjacent tips (species *i* and *j*) which have trait values *X_i_* and *X_j_*, respectively, and with common ancestor *k*, the contrast *u_ij_* = *Xi* – *Xj* is computed. This value has expectation zero and variance *V_i_* = *v_i_* + *v_j_*, where *v_i_* and *v_j_* are the lengths of the branches leading to nodes i and j respectively.

Second, we assign *k* the character state

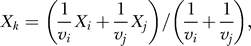
i.e., the variance weighted mean of the two species observations.

Third, to account for the statistical uncertainty involved in estimating *X_k_*, the node below *k* is increased from *v_k_* to 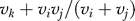 .

Fourth, the two tips are removed from the tree, leaving *k* as a tip, and the process is repeated until all tips on the tree have been removed.

The final node (i.e., the root) will have a zero contrast, by definition, but has a variance, which is the error in the ancestral state at the root, accumulated throughout the tree. Contrasts are calculated for each trait under study and are then correlated to statistically test for correlated evolution between characters.

The usual test of the adequacy of the Brownian model is to test for a significant correlation between the standardised contrasts and their expected standard deviations [17]. Under Brownian motion, there should be no correlation, and a significant correlation indicates a significant departure. As we show below, this test is generally only moderately effective or ineffective in testing for departures from Brownian trait evolution under the niche-filling model outlined above (note that the performance of this test for niche-filling models is erroneously reported in [10] as being very good, owing to a programming error). More generally, this procedure does not explicitly test whether the rate of trait evolution is declining in accord with what is expected in an adaptive radiation.

### Node Height Test

As a test of whether contrasts vary in a manner consistent with niche-filling models, here we correlated phylogenetic contrasts with the heights of the nodes at which they are generated [18]. The height of a node is defined as the absolute distance between the root and the most recent common ancestor of the pair from which the contrast is generated. A significant relationship between these indicates that the rate of trait evolution is changing systematically through the tree, compatible with the prediction that the amount of trait evolution is dependent on the number of species present in an adaptive radiation [18]. This test requires that (i) a dated tree with branch lengths is available and (ii) that the order of origin of nodes in the tree is correct. If either of these conditions is not met, then the test will not work. The test described in the next section does not require either assumption.

### Randomisation Test

In this section, we outline a simple randomisation test for whether data are consistent with Brownian motion. As noted above, the size of a standardised contrast should be independent of its position on the phylogeny, i.e., small and large values should be equally as likely to occur on any part of the tree. This is in contrast with the niche models, where the amount of change in phenotype between a parent and daughter species depends on the position of the parent species in niche space as well as the number of species present at that time. Therefore a further test of the adequacy of the Brownian model that we propose is to use the observed values of the standardised contrasts in order to generate random datasets and to test whether these have similar statistical properties to the data: under the Brownian model, randomly generated data should not differ significantly from the original data.

The procedure runs as follows: (i) using the tree and original data, the trait variance (and covariance if several traits are being studied) is calculated; (ii) standardised contrasts are estimated; (iii) the standardised contrasts are randomised on the tree (see below), with the trait variance and covariance estimated for each pseudoreplicate; and (iv) the distribution of the variances of the pseudoreplicates is examined to determine whether the distribution of the randomised data encompasses the observed values, and the probability of the data is determined. Specifically, the randomisation is then performed in the following way.

First, the trait value (*X*_0_) is set at the root node.

Second, of the *n* – 1 standardised contrasts (calculated as outlined above) one (*u_i_*) is chosen at random. If two traits are studied, then a pair is chosen, the pair having been taken from the same node on the original tree.

Third, this contrast is used to reconstruct character states. The two branches (*j* and *k*) subtended from the root node have lengths *v_j_* and *v*_k_, respectively. By definition, if evolution is Brownian, then

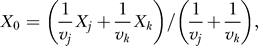
where *X*_j_ and *X_k_* are the trait values at nodes *j* and *k,* respectively, and are to be estimated. Then, using the randomly chosen contrast, if evolution is Brownian, the following second relation can be used to generate random values for *X_k_* and *X*_k_: 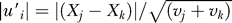 , and the sign (positive or negative) of each side of this second equation is assigned at random.

The second and third steps are then repeated until all nodes have been assigned randomised values, including the tips. The key point about this analysis is that under the Brownian model, any of the values of the *u*'s could have been measured at any of the nodes on the tree.

We used 1,000 randomised datasets to estimate empirical confidence intervals for the trait variance (and covariance in the case of two traits) measured at the tips for each dataset studied. Under the Brownian model, the randomised distribution should include the observed data, whereas under non-Brownian models, the observed data may yield values different from the randomised values.

## Results

We report below the proportion of simulated trees for which the randomisation test rejects Brownian motion. When data were simulated according to Brownian motion, we found that this proportion of trees for which the tests rejected the Brownian model was the expected 5%. For simplicity of presentation, therefore, in the results below, the rejection rate of the Brownian model is represented by the dotted line at the level of *p* = 0.05.

### Single Traits

**Figure 1:**
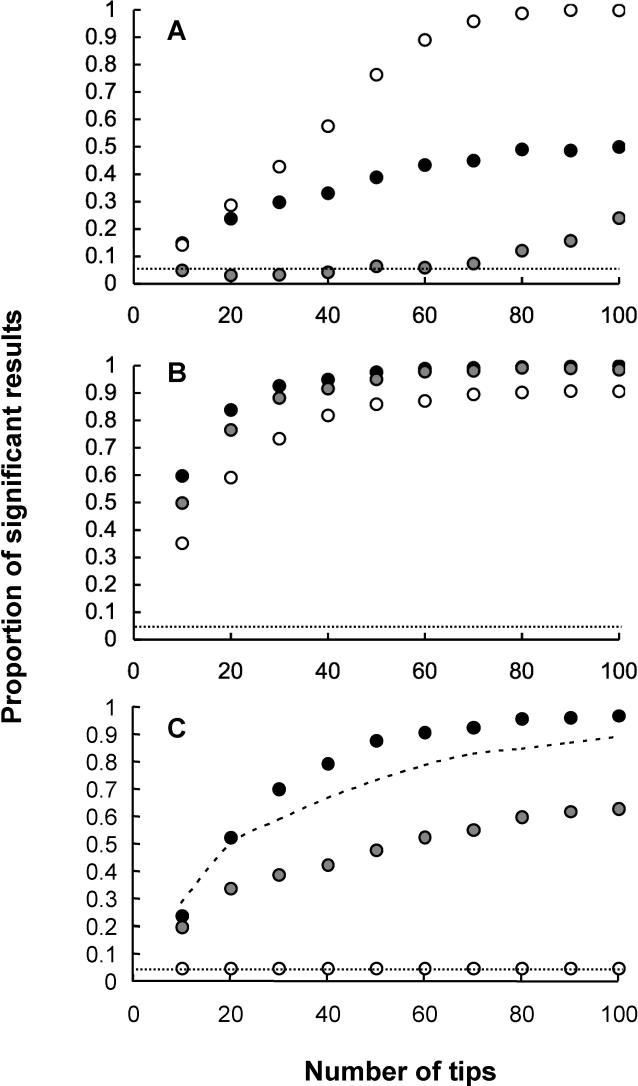
Analysis of Simulated Data from the Niche-Filling Model of Adaptive Radiation and Brownian Models Using the Tests Described in the Text The tests are applied to the variance in traits that have evolved singly. The graphs show the proportion of significant results obtained from the test applied, which, in the case of the Brownian model, is the type I error rate of the test and is represented by the dashed line. The data points show the power of the tests to reject the Brownian model when data were generated according to the niche-filling model. The mode of speciation was varied as follows: black circle, decelerating net rate of speciation; gray circle, constant net rate of speciation; white circle, increasing net rate of speciation. (A) Standard diagnostic (correlation of absolute values of standardised contrasts with expected standard deviations). (B) Correlation of absolute values of standardised contrasts with node heights. (C) Randomisation test. In (C), the dashed line is the randomisation test, applied without using branch length information.

Figure 1A shows the performance of the standard diagnostic [17] applied to data from single traits evolved under the niche-filling model. For data generated on trees evolving according to a Yule process, the test performs reasonably well. However, for trees generated assuming constant or declining rates of speciation, the test performed poorly. This is hardly surprising, because this test is essentially intended to reveal a simple speciational mode of trait evolution rather than systematic changes in evolutionary rates.

Correlation of node heights with absolute values of contrasts is a more reliable indicator of deviation from the Brownian model for single traits evolved under the niche-filling model (Figure 1B). This is unsurprising, because this test is intended to reveal changes in the rate of trait evolution through time. The power of this test is generally high, even for relatively small phylogenies (10 tips). The power of this test is, to some degree, also affected by variation in tree structure resulting from different models of speciation. However, this effect is far less marked than for the standard test in Figure 1A.

Figure 1C shows the application of the randomisation test to single traits evolving according to the niche-filling model. The power of the randomisation test to detect non-Brownian trait evolution depends on the mode of speciation. In the case of the constant and decelerating rates of speciation, the power of the test to reject Brownian motion is generally acceptable. However, the power of the test is lower when speciation proceeds according to the Yule process. This is because the randomisation test works by computing contrasts as the change in trait value divided by the square root of path lengths. In the niche-filling model, the change in trait value per speciation event declines moving from the root of the tree to the tips. Under the Yule process, the per lineage rate of speciation is constant. Because the number of lineages increases through time, the net rate of speciation (i.e., the number of speciation events occurring per unit time) increases as the number of species increases moving from the root to the tips. This has the consequence that branch lengths decline moving through nodes from the root to the tips. Thus, both trait values and branch lengths decline simultaneously, if the niche-filling model is applied with the Yule model of speciation. Therefore the test is effectively confounded and its power compromised.

The topology of phylogenies generated under the niche-filling model should be unaffected by the choice of model for speciation: it is only the distribution of branch lengths that is affected by this component of the model. Consequently, we examined the power of the randomisation test ignoring branch lengths information (i.e., with all branch lengths set to one). The result of doing this is shown by the dashed line in Figure 1C. Ignoring branch lengths, the test has acceptable power, which, of course, is unaffected by the mode of speciation. Consequently this provides a robust counterpart to the randomisation or node-height correlation analysis.

### Correlated Traits

**Figure 2:**
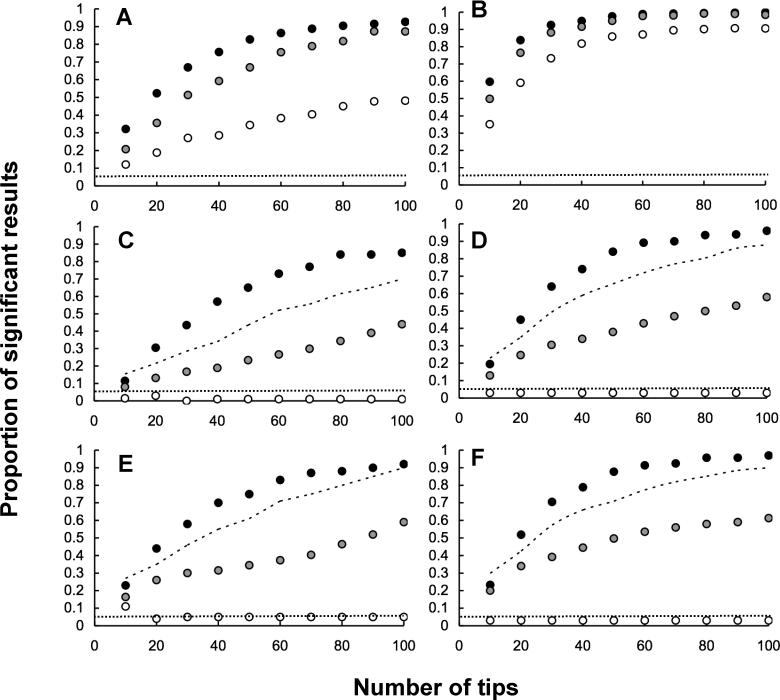
Analysis of Simulated Data from the Niche-Filling Model of Adaptive Radiation and Brownian Models Using the Tests Described in the Text The tests are applied to data on the correlated evolution of two traits. The graphs show the proportion of significant results obtained from the test applied, which, in the case of the Brownian model, is the type I error rate of the test and is represented by the dashed line. The data points show the power of the tests to reject the Brownian model when data were generated according to the niche-filling model. The mode of speciation was varied as follows: black circles, decelerating net rate of speciation; gray circles, constant net rate of speciation; white circles, increasing net rate of speciation. (A and B) node height test; (C and D) randomisation test applied to data on trait variances. (E and F) randomisation test applied to data on trait co-variances. The strength of association between traits was set at *r* ^2^ = 0.5 (A, C, and E) or *r* ^2^ = 0.9 (B, D, and F) . In (C) and (F), the dashed line is the randomisation test, applied without using branch length information.

We analysed the performance of the node height correlation and randomisation test for traits with a moderate (*r* ^2^ = 0.5) and strong correlation (*r* ^2^ = 0.9). Under speciation according to the Yule process, the performance of the node-height correlation was depressed for moderately correlated traits (Figure 2A), although it was high when the correlation between traits is strong (Figure 2B).

In the case of two characters, we used the randomisation approach to generate sampling distributions for both the variance in each trait as well as the covariance between traits. The power of the randomisation test to detect non-Brownian trait evolution through analyses of trait variance is affected by the model of speciation, especially in the case of the Yule process, again because the test is confounded under this model of speciation and trait evolution (Figure 2C and 2D). The ability of the test to detect non-Brownian covariance of traits across species under the niche-filling model is only slightly affected by the trait correlation (compare Figure 2E and 2F).

In general, it would be expected that the randomisation test would perform best when analysing traits that evolve closely together. In terms of statistical analyses (as opposed to distinguishing models of evolution), this is unlikely to be an issue, because it has been shown that comparative methods tend to be most compromised by the niche-filling model when the correlation between traits is high [10].

As in the case of single traits, by ignoring branch lengths, the compromise in the power of tests resulting from confounding effects of the branch-length distribution resulting from different models of speciation can be overcome. As shown in Figures 2C–2F, the power of the randomisation test applied ignoring branch lengths is acceptable. Again, these results indicate that this provides a potentially robust complement to the node height correlations.

In summary, the power of the two tests to detect non-Brownian evolution depends on the model of speciation as well as the correlation between traits. In both cases, by assuming a model of speciation that produced a distribution of branch lengths that exactly matched the pattern of accumulation of variance in traits, it was possible to confound the test and hence decrease power. However, by ignoring branch lengths and applying the randomisation approach, it is possible to deal with this problem.

### Examples

Here we discuss two case studies from classic adaptive radiations. In both cases, independent morphological and phylogenetic information are available. The aim here is to show how the tests we have described may be applied to deduce the existence of non-Brownian modes of trait evolution.

#### Old World Leaf Warblers.

We used the tests to analyse trait evolution in Old World Leaf warblers (Phylloscopus spp.). Richman and Price [19] published data on feeding preferences in these species and analysed two components of related to feeding ecology: body size and prey size. They also presented a phylogeny for the species and used this to conduct a phylogenetically corrected analysis of the correlation between these two characters.

**Figure 3:**
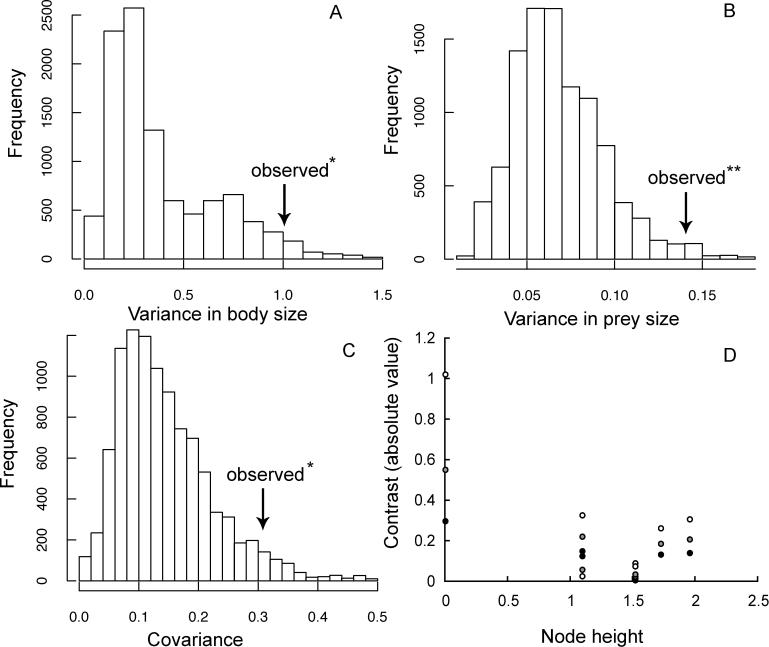
Analysis of Data on Evolution of Feeding Adaptations in New World Leaf Warblers The histograms show the distribution of randomised values of variance in (A) body size and (B) prey size, as well as (C) randomised values of the covariance between body size and prey size, using the randomisation procedure described in the text. The observed values are indicated by arrows and in each case, lie significantly to the right of the randomised values indicating deviation from the Brownian model. (D) The relationship between contrast values (absolute) and node heights. The symbols show correlations for contrasts in body size (black circles; *r* = −0.758; *p* = 0.002), prey size (gray circles; *r* = −0.719; *p* = 0.027), and the square root of the product of the two (white circles; *r* = −0.853; *p* = 0.004). Note *p* values are one-tailed, testing for a reduction in the rate of trait evolution, as in the niche-filling model. Single asterisk signifies *p <* 0.05. Double asterisk signifies *p* < 0.01.

Figure 3 shows the randomisation test applied to the data. It is clear that the test rejects the Brownian model for both of the characters singly (Figure 3A and 3B), as well as for the correlation between them (Figure 3C). Finally, as shown in Figure 3D, there is a significant decline in the value of the standardised contrasts with node height. As indicated in the figure legend, the correlations are statistically significant, in accord with the results of the randomisation tests. In this example, the standard diagnostic test [17] also indicates substantial deviation from the Brownian model.

#### *Dendroica* Warblers.

**Figure 4:**
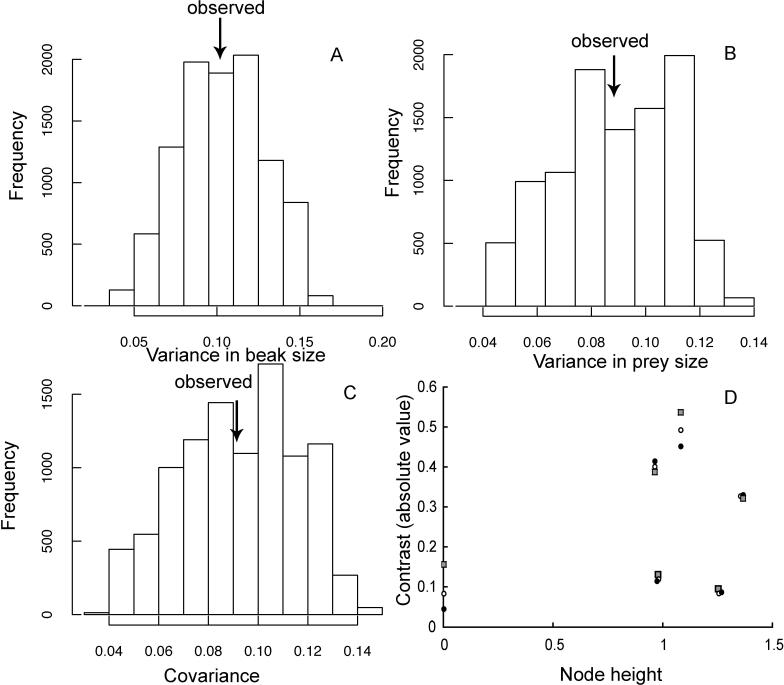
Analysis of Data on Evolution of Feeding Adaptations in *Dendroica *Warblers The histograms show the distribution of randomised values of variance in (A) beak size and (B) prey size, as well as (C) randomised values of the covariance between beak size and prey size, using the randomisation procedure described in the text. The observed values are indicated by arrows and in each case lie near the centre of the distribution, revealing no evidence of deviation from the Brownian model. (D) The relationship between contrast values (absolute) and node heights. The symbols show correlations for contrasts in beak size (black circles; *r* = 0.280; *p* = 0.30), prey size (gray circles; *r* = 0.494; *p* = 0.15), and the square root of the cross product of the two (white circles; *r* = 0.354; *p* = 0.24). Note *p* values are one-tailed, testing for a reduction in the rate of trait evolution, as in the niche-filling model.

We analysed data from a similar radiation of *Dendroica* warblers (data taken from [20]). The data are measurements of bill length and bill depth for seven species. As shown in Figure 4A–4C, the distributions of randomised variances are in accord with the observed values. Moreover, the relationships between node height and absolute contrast values are nonsignificant (Figure 4D) and do not fail the standard diagnostic test [17]. Admittedly, this is again a small grouping of species, the main point being that the comparison of Figures 3 and 4 shows how the difference between modes of evolution might be diagnosed.

## Discussion

It was previously shown that the model proposed by Price [9] was incapable of being transformed to a Brownian model, and as a consequence, inferences drawn from analyses assuming such a model would be incorrect [10]. The key results presented above are as follows: (i) some niche-filling models may be readily distinguished from Brownian models using simple diagnostic tests; (ii) these are generally powerful, even for small datasets, although the possibility of confounding has to be considered carefully; and (iii) we show how the approach might be applied to explore patterns of evolution in similar radiations.

A range of parametric approaches exist for diagnosing phylogenetic dependence in comparative data [4,20–24], as well as for testing whether data fit the assumptions of the Brownian model [17], or for transforming data onto a scale onto which they are Brownian [3,6,7,20]. Such analyses of course presuppose that the pattern of evolution can be represented by such a model and this basic assumption may be incorrect [10]. The form of niche-filling model analysed above yields data that exhibit close phylogenetic dependence, because closely related species tend to have similar trait values. Thus, these tests of phylogenetic dependence cited above will not be capable of distinguishing the niche-filling model from Brownian motion.

The niche-filling model yields punctuated and time-varying rates of evolution; thus, appropriate diagnostics may yield important evidence of the existence of non-Brownian evolution. Harmon et al. [13] propose an analysis that examines whether the reconstructed accumulation of trait variability across species through time agrees with the Brownian model. The comparison is based on a randomisation procedure using the observed phylogeny. This test therefore is essentially examining a similar effect to the analysis proposed here. As noted above, the problem with many such tests is the likely potential for confounding resulting from the distribution of branch lengths. The randomisation proposed in [13] could presumably suffer in the same way as the randomisation proposed here does. The randomisation method outlined here, however, is relatively robust if branch length information is not used.

Many adaptive radiations contain few species (e.g., Darwin's finches, 14 species; *Dendroica* warblers, 12 species; Physcollopus warblers, 15 species; Hawaiian silverswords, 25 species), and thus techniques are required to explore such small datasets. The tests reported here have moderate-to-strong power when applied to such sample sizes, and as shown with the example datasets, the tests can be applied to small datasets.

The Brownian model of trait evolution is not incompatible with traits evolving in an adaptive manner nor subject to adaptive constraints. The key assumption is that trait change is random and the likelihood of change is unaffected by the state of a trait or by other species. This does not preclude the possibility that the Brownian model can model adaptive change in cases where species are evolving independently of each other. An important novel feature of niche-filling models is that as niche-space becomes filled, the amount of possible future change in traits tends to become less. It is this element of the evolution of traits that simple variants of the Brownian models are currently unable to capture.

It is important to emphasise that the analysis presented here is diagnostic analysis; further work is required on developing techniques for inferring deviations from Brownian motion under a range of ecological processes. Apart from niche-filling models [9,10], there have been no attempts to include explicit ecological mechanisms into models for the evolution of comparative data. Such models are potentially extremely valuable, because they will allow us to explore the detailed nature of mechanisms underlying the evolution of ecological communities.

## Supporting Information

Dataset S1 A Program (Mac OS X format), C++ Code, and Example Datasets for the Analyses Above (296 KB ZIP)
